# Characterization of 20 Oenological Tannins from Different Botanical Origins for Formulation of Blends with Redox Potential Tuning Ability in Model Wine Solution

**DOI:** 10.3390/antiox12071399

**Published:** 2023-07-07

**Authors:** Francesco Maioli, Luigi Sanarica, Lorenzo Cecchi, Bruno Zanoni, Nadia Mulinacci, Valentina Canuti

**Affiliations:** 1 Department of Agricultural, Food, Environmental, and Forestry Sciences and Technologies (DAGRI), University of Florence, Via Donizetti, 6, 50144 Florence, FI, Italy; francesco.maioli@unifi.it (F.M.); bruno.zanoni@unifi.it (B.Z.); valentina.canuti@unifi.it (V.C.); 2 Enolife S.r.l., Viale Delle Imprese s.n., 74020 Montemesola, TA, Italy; luigisanarica@enolife.it; 3 Department of NEUROFARBA, University of Florence, Via Ugo Schiff, 6, 50019 Sesto Fiorentino, FI, Italy; nadia.mulinacci@unifi.it

**Keywords:** gallotannin, polyphenols, ellagitannins, condensed tannins, proanthocyanidins, HPLC-DAD-MS, CIELab, antioxidant properties, proteins

## Abstract

Twenty oenotannins from different botanical origins were studied in model wine solution (1 g/L, 12% ethanol, pH 3.5). An original device was created for measuring Oxidation–Reduction potential (*ORp*) of the solutions at 20 °C in strict anoxic condition by the electrochemical method of the platinum electrode zero-current potential. Reactivity against proteins and antioxidant properties were related to the chemical structure and, consequently, to the botanical origin of the oenotannins. The highest turbidity after BSA addition (ΔNTU > 1000) values were measured for the gallic hydrolysable tannins. The *ORp* versus standard hydrogen electrode ranged from 420 to 260 mV. The ellagitannins had the highest antioxidant power (*AP*%), followed by condensed tannins and gallotannins, highlighting a correlation with the phenolic profile. Based on these findings, two formulations were prepared as a blend of some of the tested oenotannins, with the ability to increase (MIX1) and decrease (MIX2) the *ORp* of the model wine.

## 1. Introduction

Oenological tannins (oenotannins) are phenolic compounds with a molecular weight greater than 500 Da, which are extracted from one or more botanical species and used in winemaking for their antioxidant properties and ability in proteins precipitation. Many types of oenological tannins are present on the market, and they can be classified according to the chemical structure, the botanical origin, or the production process [1,2]. The classification in condensed and hydrolysable tannins is usually used to show the wide number of tannins with different molecular structure and functional characteristics [3]. The botanical origin is also strongly linked to the oenotannin structure; for example, phenolic compounds from grape skins and seeds, tea, quebracho and acacia belong to the condensed tannins, whereas those from chestnut, oak, myrabolan, nut galls and tara belong to the hydrolysable ones [3].

Condensed tannins, also called proanthocyanidins, are a group of polyhydroxyflavan-3-ol oligomers and polymers constituted by flavan-3-ols subunits (i.e., catechin, epicatechin, epigallocatechin, fisetinidol) linked together through C-C bonds [4]. Several types of condensed tannins exist, which are differentiated by the monomer released after acidic treatment, the level of galloylation and the degree of polymerization [1,3]. For example, the mimosa tannins are prorobinetidins, the quebracho tannins are profisetinidins with a high ramification level, the grape skin tannins are a mix of procyanidins and prodelphinidins with a high degree of polymerization and a low level of galloylation, and the grape seeds tannins are procyanidins with a high level of galloylation and a high degree of polymerization [1,5]. 

Hydrolysable tannins are usually constituted by a D-glucose unit esterified by several moieties of gallic acid and its derived forms such as ellagic acid. They can be subclassified according to the Okuda classification in gallotannins, which are type-I hydrolysable tannins with the sugar unit linked to several galloyl moieties, and ellagitannins, which are formed when inter- or intramolecular oxidative coupling between the galloyl residues occur to form C-C and C-O ether bonds [1,6,7]. 

Ellagitannins can be characterized by the hexahydroxydiphenoyl group (HHDP) (i.e., the type-II hydrolysable tannins), the dehydrohexahydroxydiphenoyl group (DHHDP) (i.e., the dehydroellagitannins or type-III hydrolysable tannins) and the chebuloyl or elaeocarpusoyl group (i.e., the modified dehydroellagitannins or type-IV hydrolysable tannins). The gallagyl esters (e.g., the punicalagins from pomegranate) and the C-glycosidic ellagitannins (e.g., the castalagin and the vescalagin from chestnut and oak) are the other types of ellagitannins [7,8,9,10,11,12,13]. 

The oenological tannins addition to musts and wines was authorized and regulated by the International Organization of Vine and Wine (OIV) [14] both to facilitate the protein stabilization and to promote the expression of color in red wines [3,15,16]. However, the use of tannins has become increasingly widespread due to their antioxidant properties [1,3,15,16,17,18,19,20,21]. Indeed, both oxygen concentration and redox potential are two critical operating conditions in the prefermentation processing of grapes, must fermentation, wine stabilization and shelf life. The oxygen affects wine’s sensory and chemical characteristics; noncontrolled oxygen exposure makes wine prone to either reductive off-flavors or oxidative spoilage, whereas the controlled exposure with suitable winemaking practices contributes to enhancing wine quality over aging [22,23]. Therefore, suitable tools to control oxygen concentration and to protect the wine against its detrimental effect are very appreciated by winemakers, and the concerns related to the side effects of sulfur dioxide are prompting an increasing search for suitable alternatives [16,24]. 

The measurement of antioxidant properties of food and beverages is often based on the reducing power or the antiradical activity of antioxidants using several assays (i.e., ABTS, DPPH, FRAP, etc.), but the characteristic of an antioxidant agent connected with its redox potential cannot be evaluated by the above methods. Then, a suitable characterization of the antioxidant properties should be also carried out using electrochemical methods, which are able to measure the oxidation or reduction state of a medium [25]. The common electrochemical methods in enology consist (i) in the zero-current potential of a platinum electrode [26,27] or (ii) in the cyclic voltammetry based on measurement of the electric current intensity due to increase/decrease of potential at 100 mV/s using glassy carbon electrodes [28,29]. Some literature data showed the antioxidant properties of the oenological tannins, which have electroactive functional groups generating an electric current when these groups are either oxidized or reduced at the electrode surface [30,31]. Vignault et al. [1] measured the chemical composition and antiradical activity of 36 commercial tannins, which showed different antioxidant properties according to their chemical structure; the ellagitannins were the most effective ones to protect wine against oxidation, followed by condensed tannins and gallotannins. Ricci et al. [32] compared the DPPH assay and the cyclic voltammetry to measure the antioxidant properties of 20 commercial tannins, and they suggested the cyclic voltammetry as a valid alternative of the DPPH assay. Since the antioxidant properties are also linked to the oxygen consumption capacity, some authors [1,33] measured the oxygen consumption rate of commercial tannins dissolved in a model wine solution; the oak tannins and, in general, the ellagitannins showed the highest ability of a fast oxygen consumption without altering the amount of sulphites in wine samples. 

Although commercial oenotannins are often blends either of different tannins or of tannins obtained from different processing extractions, literature data on antioxidant properties of the above mixtures are lacking. Therefore, in this research, twenty commercial oenological tannins from a wide spectrum of botanical origins were characterized from both chemical–physical and antioxidant properties points of view. An original device for redox potential measurement by a platinum electrode was created to this aim. The effect of the addition of the studied tannins (either in pure or mixture form) on redox potential of a model wine solution was studied. As a result, two blending formulations of the above tannins with tuning ability of redox potential were prepared and tested for oenological application. 

## 2. Materials and Methods

### 2.1. Materials

#### 2.1.1. Chemicals

Ultrapure water was prepared using the Elix 5 System (Millipore, Billerica, MA, USA). Acetonitrile of HPLC-MS grade was from Panreac (Barcelona, Spain). Bovine Serum Albumin (BSA ≥ 99.5%), 1,1-diphenyl-2-picrylhydrazyl radical (DPPH), ethanol (EtOH 99%) and L-tartaric acid of analytical reagent grade were from Sigma-Aldrich (St. Louis, MO, USA). 

#### 2.1.2. Tannins

Twenty commercial oenological tannins were provided by Enolife S.r.l. (Montemesola, TA, Italy). According to the chemical structure of the main phenolic compounds in tannins from different botanical origins, the above tannins were classified and coded as follows (Table 1):

- nine belonged to the condensed tannins (i.e., from SDS to THD codes in Table 1): two from grape seeds (proanthocyanidins including procyanidins types), two from quebracho (profisetinidins type), one from mimosa (prorobinetidins type), two from tea (catechin/epicatechin gallates types), one from prunus wood (procyanidins type) and one from citrus wood;
- eleven belonged to the hydrolysable tannins (i.e., from GAS to OEE codes in Table 1). They were gallotannins, two from nut gall and one from tara, but also ellagitannins, one from mirtacee, one from chestnut and six from oak.

Some commercial tannins from the same botanical origin were of different geographic origin; different treatments were applied to some oak tannin samples, and either one or two cycles of extraction were applied for tannin processing (Table 1). Two blending formulations of the above tannins were also prepared for improving the capability of achieving an increase (MIX1) and a decrease (MIX2) of the *ORp* in the model wine, compared to the pure oenotannins, as better explained in Section 3.5 (“Blending formulations of oenotannins”) of the paper.

#### 2.1.3. Model Wine Solution with Tannins (1 g/L Oenotannin Solutions)

A model wine solution was prepared as follows: 4 g/L of L-tartaric acid were added to a 12% EtOH in water solution. Once the above acid was completely dissolved, pH was adjusted to 3.5 with NaOH, keeping the solution under magnetic stirring. Then, a model wine solution with tannins was prepared for each of the tannin samples in pure form or in mixture as follows: 100 mg of tannin sample were dissolved in 100 mL of the above model wine solution under magnetic stirring to prepare 1 g/L solutions. The model wine solution with tannins samples (i.e., the oenotannin solutions) were centrifuged (Mikro 12–24 Centrifuge HETTICH, Tuttlingen, Germany) at 12,000 rpm for 10 min before all the analytical measurements.

### 2.2. Methods

#### 2.2.1. Coloring Properties of the Oenotannin Solutions

The coloring properties of the oenotannin solutions were defined according to OIV [14]. Visible absorbance at 420 and 520 nm of the oenotannin solutions was acquired using a 1 mm quartz cell using a UV-visible spectrophotometer Lambda 35 (Perkin Elmer Inc., Shelton, CT, USA), with the UVWinLab software used to record the data (version 2.85.04, Perkin Elmer Inc., Shelton, CT, USA). Elix 5 System water was used as a reference. Both the absorbances were used to determine the potential color contribution of oenotannin solutions to wine according to OIV [14].

#### 2.2.2. CIELab Coordinates of the Oenotannin Solutions

The CIE (Commission Internationale de l’Eclairage) L*, a* and b* color coordinates of the oenotannin solutions were measured according to OIV [34]. Visible spectra were recorded in transmittance at 400–700 nm using a 10 mm path length quartz cell and the Lambda 35 UV-Visible spectrophotometer (Perkin Elmer Inc., Shelton, CT, USA) equipped with the RSA-PE-20 Integrating Sphere accessory assembly (Labsphere, North Sutton, NH, USA). UV WinLab software was used to record the spectra (version 2.85.04, Perkin Elmer Inc., Shelton, CT, USA), and CIELab color coordinates were calculated for the CIE illuminant D65 and 10° standard observed conditions using Color software (version 3.00, 2001, Perkin Elmer Inc., Shelton, CT, USA). Color differences between the oenotannin solutions were determined using the Δ*E* value of the CIELab diagram, according to the following equation:

(1) $$\Delta E=\sqrt{{\left({\Delta L}^{*}\right)}^{2}+{\left({\Delta a}^{*}\right)}^{2}+{\left({\Delta b}^{*}\right)}^{2}}$$

When Δ*E* ≥ 3, the differences between oenotannin solutions are perceivable by the human eye [35].

#### 2.2.3. Precipitation Assay of the Oenotannin Solutions 

The ability to precipitate protein is the defining characteristic of the tannins or plant polyphenols [36]. The precipitation method was carried out as follows: 20 mL of the oenotannin solutions in model wine were placed in a nephelometric cuvette (Hanna Instruments, Smithfield, RI, USA), and 2 mL of aqueous BSA solution were added (7% *w*/*v*). The turbidity was measured both before and after BSA solution addition and monitored until turbidity stabilization (approximately 5 min) using a HACH2100N turbidimeter (Hach Lange SrL, Lainate, MI, Italy). Two ΔNTU values were determined as follows: (i) ΔNTU1 was the difference between the NTU value at the time of BSA addition and the initial value before BSA solution addition; (ii) ΔNTU2 was the difference between the NTU value registered after turbidity stabilization (after 5 min) and the initial value before BSA solution addition.

#### 2.2.4. Total Phenols Estimation

Total Phenols Estimation was measured as described by Ribereau-Gayon [37] and OIV [14]. The oenotannin solutions were diluted 1:50 with Elix 5 System water (Millipore, Billerica, MA, USA), and the ultraviolet absorbance at 280 nm (10 mm path length quartz cell) of the samples was measured on a Lambda 35 (Perkin Elmer, Shelton, CT, USA) UV-visible spectrophotometer by means of the UVWinLab software (version 2.85.04, Perkin Elmer Inc., Shelton, CT, USA). Elix 5 System water was used as a reference. Total Phenols Estimation was expressed as gallic acid equivalents/g and transformed in % as described by OIV [14].

#### 2.2.5. Phenolic Characterization by HPLC-DAD-MS Analysis

The oenotannin solutions were analyzed by HPLC-DAD-MS to define their phenolic profile. A 1260 Infinity II LC system was used, which was provided with two different detectors: a Mass Spectrometry Detector (MSD) equipped with an API-electrospray interface (InfinityLab LC/MSD) and a Diode Array Detector (DAD) (Agilent, Santa Clara, CA, USA). The molecules were separated in a Luna (150 mm × 3.0 mm, 2.7 µm, Phenomenex, Torrance, CA, USA) column. Acetonitrile (A) and H_2_O (pH 3.2 with formic acid) (B) constituted the mobile phase, and a multistep linear gradient was used, as follows: solvent A varied from 5% to 15% in the first 20 min, stayed at 15% for 5 min, then varied from 15% to 25% in 10 min, staying at 25% for 8 min, then, it passed at 100% in 5 min, staying at 100% for 4 min, and finally returning at 5% in 3 min, for a total of 55 min, followed by a reconditioning of 10 min. The flow rate was 0.8 mL/min, with an injection volume of 5 µL. Chromatograms were recorded at 240, 254, 280, 308, 330 and 350. For the Mass Spectrometry Detector, the conditions of the ESI parameters were set as follows: nitrogen flow rate was 10.5 L/min; drying gas temperature was 350 °C; nebulizer pressure was 1811 Torr; capillary voltage was 3500 V. Data were acquired in the *m/z* range 100–2000 Th in full-spectrum scan mode and in negative ionization mode; a fragmenter of 150 V was applied. The following compounds were tentatively identified using both UV and MS data: gallic acid, castalagin, (+)-catechin, (−)-epicatechin, epicatechin gallate, procyanidin, dimer and trimer procyanidin, ellagic acid, and the total polymerized fraction (tannins). For quantitative determinations, 5-point calibration curves were prepared using (+)-catechin (λ = 280, linearity range 0.011–0.800 μg, *R*^2^ = 0.9999) and gallic acid (λ = 280, linearity range 0.018–4.1 μg, *R*^2^ = 0.9999). The limit of quantification (LOQ) was estimated using the standards of (+)-catechin and gallic acid, while the limit of detection (LOD) was evaluated as 1/3 of the LOQ. The values were: for (+)-catechin, LOQ = 11 ng, LOD = 4 ng; for gallic acid, LOQ = 18 ng, LOD = 6 ng. Using the obtained calibration curves, gallic acid, castalagin, ellagic acid and the total polymerized fraction were measured at 280 nm and quantified as gallic acid equivalent at 280 nm; (+)-catechin, (−)-epicatechin, epicatechin gallate, procyanidin, dimer procyanidin and trimer procyanidin were measured at 280 nm and quantified as (+)-catechin equivalent at 280 nm.

#### 2.2.6. Antioxidant Property by DPPH• Assay

The measurement of antioxidant property was carried out according to the DPPH• (1,1-diphenyl-2-picrylhydrazyl radical) assay [38], slightly modified. The preparation of the DPPH• solution was carried out by dissolving 4 mg in 100 mL of ethanol and keeping the obtained solution at 4 °C overnight without light exposure. The DPPH• solution was stored at −20 °C; before use, it was thawed to room temperature. One mL of the DPPH• solution was added to 1 mL of the sample solution (the oenotannin solutions) and the mixture kept at room temperature. A Lambda 35 spectrophotometer (Perkin Elmer, Shelton, CT, USA) was used to measure absorption at 517 nm vs. an EtOH:H_2_O 50:50 (*v*/*v*) solution as the blank, both immediately and after 20 min. The antioxidant property of the model wine solution free from tannins was also measured following the same procedure to compare its activity with that of the oenotannin solutions. Results of antioxidant activity were expressed as antioxidant power percentage (*AP*%), using the following formula:

(2) $$AP\%=\frac{A_{t0}-A_{t20}}{A_{t0}}\times 100$$

where *A*_*t*0_ is the absorbance at time 0, and *A_t_*_20_ is the absorbance after 20 min.

#### 2.2.7. Redox Potential (*ORp*)

**Figure 1:**
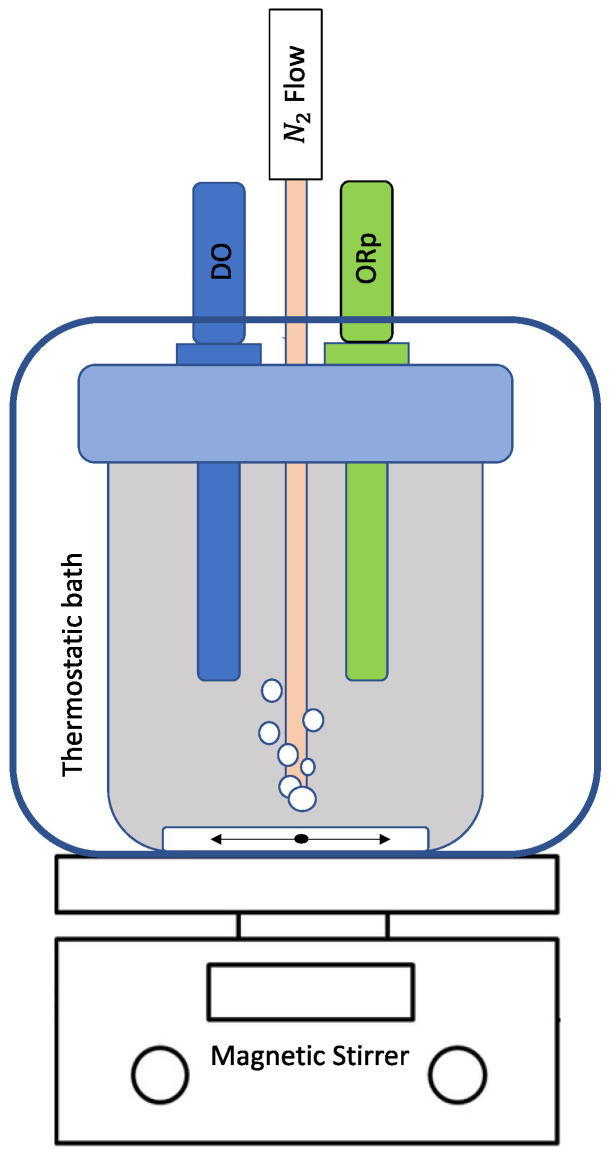
The device for *ORp* measurement of the oenotannin solutions; *ORp* = the platinum electrode combined with the temperature probe, DO = the dissolved oxygen probe, N_2_ flow = nitrogen inlet.

The measure of the oxidation–reduction state of the oenotannin solutions (i.e., the redox potential—*ORp*) was t by the electrochemical method of the zero-current potential of a platinum electrode. The official AOAC method was applied [39,40,41], and an original device was created for *ORp* measurement of the oenotannin solutions (Figure 1). In a plastic ampoule (500 mL of total volume), an Edge^®^ pH/*ORp*-meter (Hanna Instrument, Padova, Italy), equipped with an *ORp* electrode HI36180 (platinum sensor versus Ag/AgCl reference electrode in 3.5 M KCl) and combined with a temperature probe, was inserted. An optical oximeter (Oxy Level 2200, Parsec, Florence, Italy) was also inserted to measure the dissolved oxygen (DO) concentration, and a nitrogen injection system was provided for the *ORp* measurement in absence of oxygen. The above ampoule was installed inside a thermostatic bath to control the temperature (T), and the samples were continuously mixed through a magnetic stirrer. 

After a cleaning process (using the double-junction electrode cleaning solution), the *ORp* electrode was calibrated with standard redox solutions of 468 and 220 mV. All the solutions (calibration, cleaning, and storage) were purchased from Hanna Instrument (Padova, Italy). The ampoule was completely filled with 500 mL of the model wine solution, and the *ORp* measurements were carried out at 20 °C, under magnetic stirring and under nitrogen flow to strip the dissolved oxygen from the solutions; the abovementioned conditions were maintained for the whole period of measurements. Once the DO was approximately zero ppb, 1 g/L of the tannin samples in pure form or in mixture was added to the model wine solution. 

The *ORp* at time zero (*ORp*_*t0*_) was measured at the moment of tannin addition to the model wine solution, whereas the *ORp* at time t (*ORp*_t_) was measured every 15 min up to 48 h (*ORp_t48_*). T and DO values were monitored over the 48 h to check the operating conditions (i.e., T = 20 °C and DO ≈ 0 ppb). 

The *ORp* values were measured in mV versus Ag/AgCl; they were also converted in mV versus standard hydrogen electrode (SHE) using the +208 mV as conversion factor at 25 °C and pH = 3.5 [42].

### 2.3. Data Processing

Microsoft Excel was used for data collection and processing. All the chemical and physical data were expressed as mean values ± standard deviation. All the chemical analyses were performed in triplicate. Analysis of Variance followed by *F*-test and Least Significant Difference (LSD) post hoc comparison were performed using DSAASTAT software (v. 1.1, Onofri, Pisa, 2007) to identify significant differences between quantitative data. The *ORp* analysis was conducted in duplicate.

## 3. Results and Discussion

### 3.1. Basic Characteristics of the Oenotannin Solutions

The phenols content of the oenotannin solutions (Table 2) was measured as Total Phenols Estimation [14,37] (*w*/*w* % expressed as gallic acid equivalent). The gallic hydrolysable tannins showed the highest phenols content, ranging from 94.79 to 106.66% with a mean value of 102.58%; the GAD (106.66%) and TAR (106.29%) tannins were the richest ones. The ellagic hydrolysable tannins showed lower phenols contents than that of the gallic hydrolysable tannins, ranging from 16.69 to 41.36% with a mean value of 30.20%; the MIR (41.36%) and the CHS (41.28%) tannins were the richest ones. For the oak tannin samples, an effect seemed to be associated with the treatment before the tannins extraction; the dried oak tannins samples showed lower phenols contents than that of toasted and untreated oak tannins. The condensed tannins showed a lower mean value of phenols content (35.04%) than that of the gallic hydrolysable tannins, ranging from 19.72% for the MIM tannin to 46.85% for the THD tannin.

The reactivity of the oenotannin solutions to proteins was measured through the dynamic formation of turbidity after BSA addition. Information about stability and dispersion of the tannin–protein complex are important in wine stabilization, since tannins can interact with unfolded protein in wine, creating cross-linked aggregates with sulfates and phenolic compounds so as to form a visible haze [43,44]. A different behavior of turbidity occurred for the oenotannin solutions (Table 2). The TAR tannin was the most reactive to BSA with high turbidity increase immediately after protein addition (ΔNTU1 = 3328 NTU); then, the turbidity decreased at ΔNTU2 = 1823 NTU after 5 min due to a fast precipitation of the aggregates. The above trend was observed for all the gallic hydrolyzable tannins. A different behavior was observed for the SDS tannin, which formed a very high and stable turbidity value (ΔNTU1 = 2525 and ΔNTU2 = 2605 NTU), and, in general terms, for both all condensed tannins and ellagic hydrolyzable tannins. Condensed and ellagic hydrolyzable tannins formed smaller aggregates than gallic hydrolyzable tannins, creating more stable suspensions. 

The antioxidant property of the oenotannin solutions was measured by the DDPH assay, since it was able to show both mechanisms of hydrogen atom transfer (HAT) and single-electron transfer (SET) [25]. All the solutions showed a general high antioxidant power value (Table 2), ranging from 90.10% for the CIT tannin extract from citrus wood to 95.96% for the TAR tannin extract from tara. No differences due to the chemical structure were highlighted, although all tested gallic hydrolyzable tannins showed the highest values of antioxidant power, probably due to their high content in phenolic compounds (see the related Total Phenols Estimation values in Table 2).

### 3.2. Colorimetric Characteristics of the Oenotannin Solutions

The oenotannins usually show different shades of color ranging between light yellow and deep red according to the botanical origin and the extraction processing. Therefore, they can affect the color of white musts and wines if the oenotannin color is very intense. According to OIV resolution [14], use of the oenological tannins can change the wine color, depending on their inherent coloring properties. The absorbance at 420 nm allows for evaluating the yellow coloring property, whereas the absorbance at 520 nm allows for evaluating the red coloring property. According to OIV [14], oenotannins are considered a coloring agent when the absorbance at 520 nm is higher (i.e., >0.05) than that at 420 nm. All tested oenotannin solutions did not affect the red coloring property showing the absorbance at 520 nm (*A_520_*) lower than the absorbance at 420 nm (*A_420_*) (Table 2). The *A_520_* values ranged from 0.001 (the GAS tannin) to 0.134 (the OAN tannin); the oak tested tannins, the CHS and the THS tannins showed the above highest values. The *A_420_* values ranged from 0 (the GAS tannins) to 0.0759 (the OAN tannin); the GAS, the GAD and the TAR tannins showed the lowest values in absorbance at 420 nm as well as at 520 nm. The oak tested tannins showed the highest effect to yellow in the model wine, just as the CHS, the THS, the PRU and the CIT tannins, but the yellow effect was lower than 1.5, indicating that they are not a coloring agent according to OIV [14]. 

The oenotannin solutions were also tested for the CIELab coordinates measurement (Table 2). All above solutions were characterized by low values of the redness index a* (from −0.43 to 0.32) and high values of the lightness index L* (from 98.05 to 100.46). The yellowness index b* showed high variation between the oenotannin solutions (from −1.49 to 3.26); high b* values characterized solutions at an intense yellow-brown color, whereas low b* values characterized solutions with very poor color contribution. All tested solutions were in the yellow-to-red CIELab space. The highest b* values were measured in the QBS, the PRU and the CIT tannins solutions (3.26, 3.25 and 3.15, respectively), followed by the OFE, the MIR, the OAN and the SDS oenotannin solutions (2.63, 2.55, 2.43 and 2.33, respectively). The lowest b* values were measured in the GAS, the GAD, the TAR, the CHS and the THD oenotannin solutions (−1.49, −0.30, −0.01, 0.24, 0.83, respectively). The ΔE coefficient was consequently determined (Table 2). Only the QBS, the PRU and the CIT oenotannin solutions reached a ΔE ≥ 3 consistently with the above b* values, that is, the color differences were perceivable by the human eye [35,45].

### 3.3. Phenolic Characterization by HPLC-DAD-MS Analysis

The phenolic compounds already listed in Section 2.2.5 and reported in Table 3 were identified using UV and MS spectra and literature data [46,47]. The gallic acid was measured in all tested solutions, and the highest values were in the gallic hydrolyzable tannin solutions (mean value = 61.17 mg/g). The castalagin was measured both in the ellagic hydrolyzable tannin solutions (except for the MIR solutions) and in the condensed tannin PRU and CIT solutions; the CHS and the OEE solutions had the highest value of castalagin (24.90 mg/g and 25.03 mg/g, respectively); the ellagic acid values showed a quite similar behavior to the castalagin values. The flavonoids ((+)-catechin, (−)-epicatechin, epicatechin gallate, procyanidin, dimer and trimer procyanidin) characterized the phenolic profile of the condensed tannins according to botanical origin and extraction processing of tannins. The grape seed tannins (the SDS and the SDD solutions) had the highest values of flavonoids, except for the tea tannins at two extraction cycles (the THD solutions) which had the highest values of epicatechin gallate. The highest values of polymers were present in the gallic hydrolyzable tannin solutions (mean value = 185.72 mg/g).

### 3.4. The Redox Potential (ORp)

The *ORp* measurement by the electrochemical method of the zero-current potential of a platinum electrode requires that the tested system is at equilibrium to measure the potential of redox species in solutions directly at the inert electrode [29]; the above equilibrium can be reached after a long time, if the solutions contain redox pairs interconnected by nonreversible or very slow reactions at the electrode surface [48]. Therefore, in this study, the experimental curves of *ORp* against time were measured on the oenotannin solutions up to 48 h in order to reach a clear asymptotic value of *ORp*. Moreover, the use of a model wine solution in strict anoxia conditions allowed us to characterize the oxidation–reduction state of the oenotannin solutions without the interference of common wine compounds, such as phenolic and volatile compounds and dissolved oxygen. 

**Figure 2:**
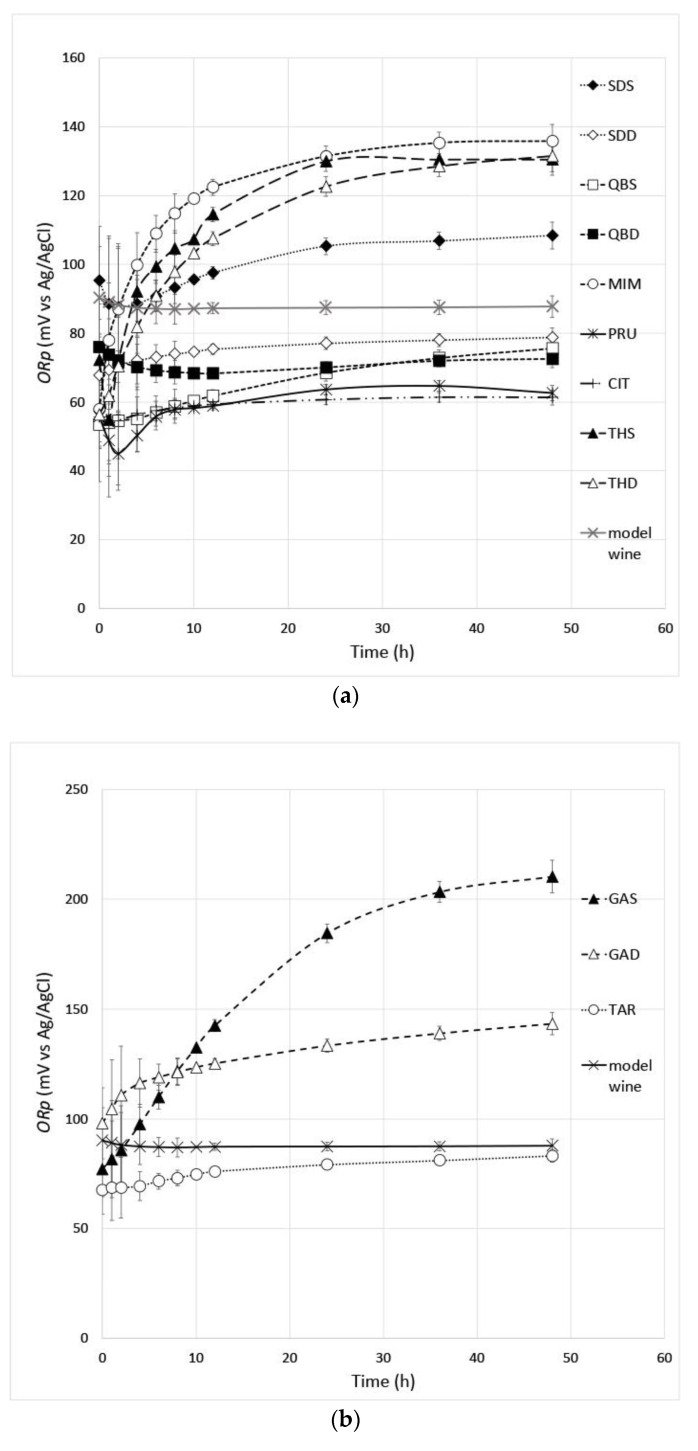

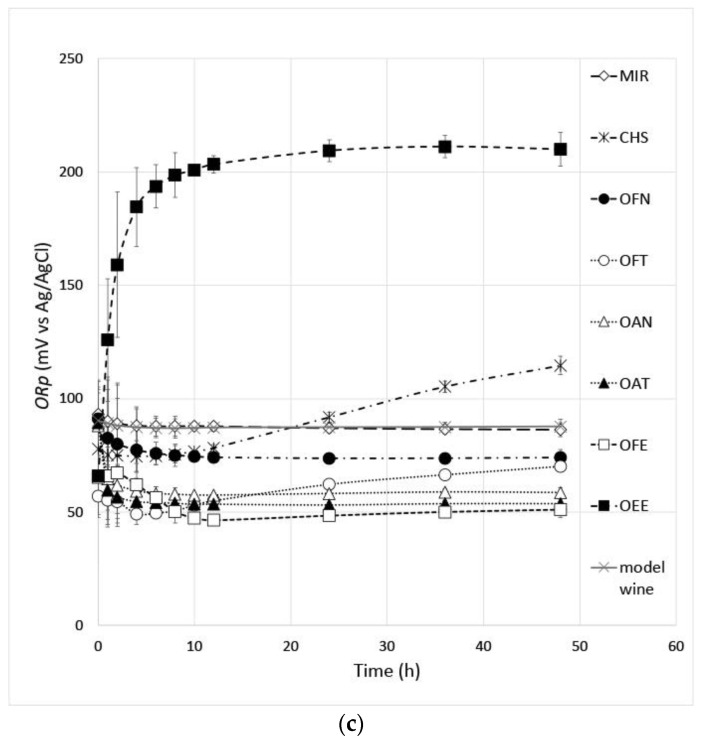
Experimental curves of *ORp* against time of condensed tannins—(**a**), gallic hydrolyzable tannins—(**b**) and ellagic hydrolyzable tannins (**c**). Error bars show the measurement standard deviations.

All experimental curves of *ORp* against time of the oenotannin solutions reached an equilibrium state (Figure 2), and the asymptotic values of *ORp* at 48 h (*ORp_t48_*) were measured (Table 4). In the oenotannin solutions, the *ORp* versus SHE values ranged from 418.3 mV to 259.1 mV, showing a different behavior to change the redox potential of the wine model in relation to the mixed redox pairs of tested oenotannins (Table 4). In general agreement with the literature data [1,32,33], the ellagic hydrolysable tannins had the highest antioxidant power, which is the best property to enrich the model wine of reducing redox pairs (see in Table 4 the lowest *ORp* values and the highest decrease of wine model *ORp* values of the tested ellagitannins), followed by the tested condensed tannins and gallotannins. Some effects of the phenolic profile of the oenotannin solutions can be also hypothesized, reflecting botanical origin and processing extraction as follows (Table 3). For example, the limited antioxidant power of tested gallotannins could be due to a phenolic profile which is only characterized by mixed redox pairs of gallic acid and polymers at high *ORp* values. Within the tested ellagitannins group, the peculiar less antioxidant behavior of the CHS and the OEE oenotannin solutions could be explained by the highest castalagin and gallic acid amounts and the lowest ellagic acid amounts due to the extraction processing [32]. The high antioxidant power of the tested condensed tannins could reflect a phenolic profile where tannins are a mixture of highly reducing compounds such as catechin and epicatechin [33]. However, within the tested condensed tannins group, further research is required to understand the peculiar less antioxidant behavior of the SDS, the MIM, the THS and the THD oenotannin solutions.

### 3.5. Blending Formulations of Oenotannins

Redox reactions qualify the winemaking process, and several changes of the redox potential occur in both musts and wines [49]. The redox potential primarily depends on the winemaking practices, which involve aeration and the related concentration of dissolved oxygen [50]. However, redox potential changes can be also obtained through processing aids such as blends of oenotannins, thus preventing must or wine oxidation due to aeration. For example, a decrease in redox potential can be useful in wines at an oxidized state in which reductants such as polyphenols, ascorbic acid and sulfite react [51]. Conversely, an increase of redox potential can be useful in wines at a reduced state in which a series of off-flavors related to an array of sulfur compounds occur [52]. 

Therefore, two blending formulations of the tested oenotannins were prepared to have different ability to tune the *ORp* of model wine with improved performance compared to the pure form of the tested oenotannins. The design concept established selection of oenotannins in relation with the discussed characteristics in the previous sections of the paper. Then, the selected oenotannins were blended to prepare 1 g/L oenotannin solutions, which were able to reach an increase (i.e., the MIX 1 oenotannin solution) or a decrease (i.e., the MIX 2 oenotannin solution) of the *ORp* in the model wine to have a redox system with lower or higher reducing power, respectively. In the MIX 1 solution, some oenotannins with the highest measured *ORp_t48_* (Table 4) were selected, whereas in the MIX 2 solution, some oenotannins with the lowest measured *ORp_t48_* were selected; the relevant quali-quantitative formulations of the oenotannin solutions were evaluated confidential from the supplier (Enolife S.r.l., Montemesola, TA, Italy).

**Figure 3:**
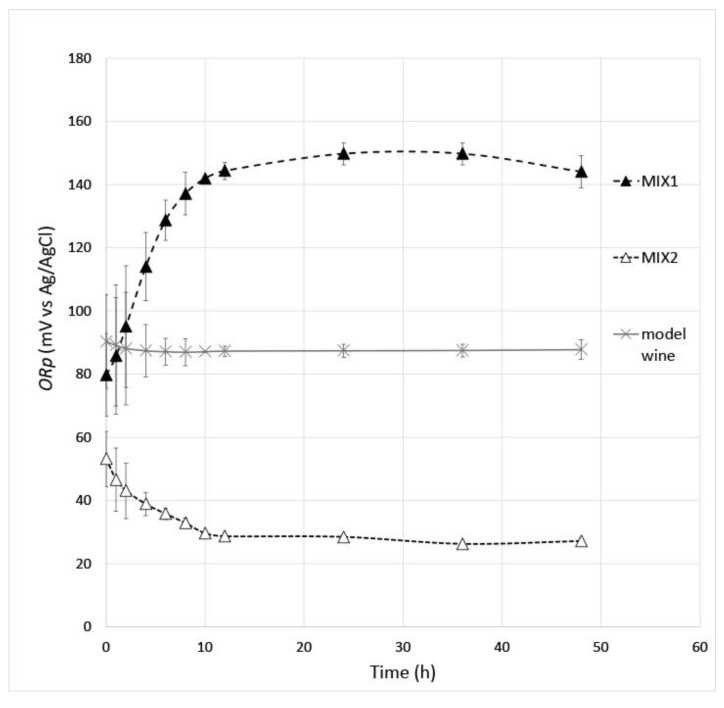
Experimental curves of *ORp* against time of the MIX 1 and MIX 2 oenotannin solutions compared to the model wine. Error bars show the measurement standard deviations.

The experimental curves of *ORp* against time of the MIX 1 and MIX 2 solutions (Figure 3) show that the designed tuning ability of redox potential in model wine solution was obtained. The MIX 1 oenotannin solution was able to increase the redox potential of the model wine (+56.2 mV) reaching an asymptotic value of *ORp_t48_* at 144.0 mV (Table 5). Instead, the MIX 2 oenotannin solution was able to decrease the redox potential of the model wine (−60.6 mV) reaching an asymptotic value of *ORp_t48_* at 27.2 mV (Table 5). The phenolic profile of the MIX 1 and the MIX 2 solutions (Table 6) can be related to the above *ORp* values in agreement with what was observed in Section 3.4 of paper. The high gallic acid and castalagin amounts combined with the low catechin, epicatechin and epicatechin gallate values could be explained by the lower reducing power of the MIX 1 solution than that of the MIX 2 solution. In the end, the MIX1 and the MIX 2 solutions showed different performances on both the reactivity to proteins and the color characteristics (Table 5). The MIX 1 solution had the highest reactivity against proteins, as it could be observed by the turbidity values, but a different color perception by the human eye (ΔE ≥ 3) occurred.

## 4. Conclusions

The obtained findings allowed a better understanding of the characteristics of oenotannins for a more precise use in winemaking. The chemical–physical characterization combined with measurement of antioxidant properties in model wine solutions was able to understand the different reactivity of the oenotannins according to their chemical structure and botanical origin. Measurement of *ORp* by the electrochemical method of the zero-current potential of a platinum electrode was able to better discriminate the antioxidant properties of oenotannins than the DPPH• assay. The above characterization can improve the suitable use of the oenotannins in winemaking. For example, oenotannins with combined properties of high reducing power-high reactivity to proteins-low coloring effect could be useful during white wine processing, where protein stability needs to be achieved, and protection from oxidation is necessary without modifying the color of wine. Moreover, the selection of oenotannins in relation to their characteristics in model wine solutions resulted in a design method to prepare commercial blending formulations of oenotannins. The present study was carried out in model wine solution to standardize the conditions; the main limit of this approach is that the interaction with the compounds present in real wine such as polyphenols, elementals, organic acids and other constituents are not considered. For this reason, further research should be carried out in real wines to test and confirm the tuning redox potential capacity based on the characterization of oenotannins in pure form.

## Figures and Tables

**Table 1:** Origin, processing treatment and structure of the tested oenological tannins.

Tannin Code	Botanical Origin	Geographical Origin	Structure of Tannin	Extraction Cycles	Treatment
SDS	Grape Seed (*Vitis vinifera* L.)	French	Condensed	1	-
SDD	Grape Seed (*Vitis vinifera* L.)	French	Condensed	2	-
QBS	Quebracho	Argentina	Condensed	1	-
QBD	Quebracho	Argentina	Condensed	2	-
MIM	Mimosa wood	Brazil	Condensed	1	-
PRU	Prunus wood	Unknown	Condensed	1	-
CIT	Citrus wood	Unknown	Condensed	1	-
THS	Tea (*Camelia sinensis* L.)	Unknown	Condensed	1	-
THD	Tea (*Camelia sinensis* L.)	Unknown	Condensed	2	-
GAS	Nut gall (Quercus)	Turkey	Gallic hydrolysable	1	-
GAD	Nut gall (Quercus)	Turkey	Gallic hydrolysable	1	-
TAR	Tara (*Caesalpinia spinosa* L.)	Peru	Gallic hydrolysable	1	-
MIR	Mirtacee (*Myrtus communis* L.)	Spain	Ellagic hydrolysable	1	-
CHS	Chestnut wood (*Castanea sativa*)	Unknown	Ellagic hydrolysable	1	-
OFN	Oak wood (*Quercus petraea* L.)	French	Ellagic hydrolysable	1	-
OFT	Oak wood (*Quercus petraea* L.)	French	Ellagic hydrolysable	1	Toasted
OAN	Oak wood (*Quercus alba* L.)	U.S.A.	Ellagic hydrolysable	1	-
OAT	Oak wood (*Quercus alba* L.)	U.S.A.	Ellagic hydrolysable	1	Toasted
OFE	Oak wood (*Quercus petraea* L.)	French	Ellagic hydrolysable	1	Dried
OEE	Oak wood (*Quercus petraea* L.)	Croatia	Ellagic hydrolysable	1	Dried

**Table 2:** Characteristics (mean ± standard deviation) of the tested oenotannin solutions; *AP* = antioxidant property by DPPH• assay; *A_420_* and *A_520_* = absorbance at 420 and 520 nm, respectively; L*, a*, b* and Δ*E* = CIELab coordinates.

Tannin Code	Total Phenols Estimation (*w*/*w* %) ^1^	Turbidity (Δ*NTU*_1_) ^2^	Turbidity (Δ*NTU*_2_) ^2^	*AP* *%*	*A_420_*	*A_520_*	L*	a*	b*	Δ*E*
SDS	37.55 ± 0.01	2525 ± 6	2605 ± 4	93.11 ± 0.13	0.158 ± 0.001	0.071 ± 0.001	99.08 ± 0.05	0.28 ± 0.06	2.33 ± 0.22	2.42
SDD	35.18 ± 0.01	210 ± 1	149 ± 6	93.29 ± 0.01	0.102 ± 0.001	0.042 ± 0.000	99.93 ± 0.12	0.09 ± 0.14	1.05 ± 0.53	0.88
QBS	31.80 ± 0.02	574 ± 3	451 ± 3	90.99 ± 0.68	0.177 ± 0.001	0.086 ± 0.000	98.63 ± 0.04	0.32 ± 0.11	3.26 ± 0.34	3.45
QBD	41.07 ± 0.02	596 ± 3	505 ± 4	92.61 ± 0.14	0.136 ± 0.000	0.079 ± 0.001	99.28 ± 0.06	0.18 ± 0.05	2.25 ± 0.26	2.24
MIM	19.72 ± 0.01	512 ± 4	583 ± 4	94.24 ± 0.24	0.077 ± 0.002	0.028 ± 0.001	99.02 ± 0.05	0.04 ± 0.02	2.22 ± 0.11	2.36
PRU	34.71 ± 0.01	566 ± 3	609 ± 5	93.89 ± 0.39	0.130 ± 0.001	0.072 ± 0.002	99.22 ± 0.04	0.01 ± 0.03	2.55 ± 0.12	3.01
CIT	41.06 ± 0.01	995 ± 5	915 ± 8	90.10 ± 0.11	0.178 ± 0.001	0.086 ± 0.001	99.36 ± 0.02	−0.28 ± 0.03	2.23 ± 0.14	3.72
THS	27.42 ± 0.01	36 ± 1	27 ± 62	94.73 ± 0.01	0.320 ± 0.000	0.104 ± 0.000	99.88 ± 0.04	−0.20 ± 0.06	1.53 ± 0.22	2.24
THD	46.85 ± 0.02	20 ± 1	7 ± 0	95.50 ± 0.07	0.380 ± 0.001	0.134 ± 0.001	99.26 ± 0.03	−0.26 ± 0.04	2.42 ± 0.18	0.64
GAS	94.79 ± 0.02	1526 ± 6	1008 ± 7	95.81 ± 0.06	0.308 ± 0.001	0.110 ± 0.001	99.81 ± 0.02	−0.27 ± 0.06	1.6 ± 0.16	1.91
GAD	106.66 ± 0.05	1430 ± 7	1108 ± 7	95.75 ± 0.10	0.221 ± 0.001	0.106 ± 0.000	99.87 ± 0.02	0.04 ± 0.08	0.24 ± 0.19	0.83
TAR	106.29 ± 0.03	3328 ± 11	1823 ± 9	95.96 ± 0.38	0.001 ± 0.000	0.001 ± 0.001	100.46 ± 0.05	0.21 ± 0.04	−1.49 ± 0.14	0.61
MIR	41.36 ± 0.04	571 ± 3	404 ± 6	92.57 ± 0.09	0.015 ± 0.001	0.004 ± 0.001	100.14 ± 0.45	−0.25 ± 0.53	−0.30 ± 1.28	2.52
CHS	41.28 ± 0.01	699 ± 3	697 ± 7	94.91 ± 0.33	0.012 ± 0.000	0.007 ± 0.001	100.10 ± 0.05	−0.10 ± 0.05	−0.00 ± 0.17	0.67
OFN	25.96 ± 0.02	285 ± 2	261 ± 3	93.90 ± 0.01	0.202 ± 0.000	0.114 ± 0.001	99.30 ± 0.04	−0.20 ± 0.05	2.28 ± 0.19	2.16
OFT	34.20 ± 0.01	181 ± 2	204 ± 2	94.42 ± 0.13	0.011 ± 0.001	0.001 ± 0.000	100.10 ± 0.03	−0.12 ± 0.04	0.83 ± 0.11	1.31
OAN	28.86 ± 0.01	215 ± 6	259 ±3	93.86 ± 0.86	0.212 ± 0.001	0.060 ± 0.001	100.06 ± 0.05	−0.39 ± 0.06	3.25 ± 0.17	2.40
OAT	30.74 ± 0.02	119 ± 3	106 ± 2	94.48 ± 0.13	0.243 ± 0.002	0.080 ± 0.003	98.05 ± 0.03	−0.43 ± 0.02	3.15 ± 0.04	1.42
OFE	16.69 ± 0.01	528 ± 4	588 ± 7	94.36 ± 0.01	0.264 ± 0.001	0.098 ± 0.003	99.05 ± 0.02	−0.26 ± 0.02	2.63 ± 0.05	2.69
OEE	22.52 ± 0.01	1010 ± 5	930 ± 9	94.82 ± 0.01	0.162 ± 0.001	0.045 ± 0.002	99.41 ± 0.02	−0.28 ± 0.01	1.84 ± 0.02	1.84
Model wine	-	-	-	-	-	-	100.52 ± 0.05	−0.02 ± 0.03	0.40 ± 0.01	-

^1^ expressed as gallic acid equivalent/g; ^2^ Δ*NTU*_1_ = difference between the NTU value at the time of BSA addition and the initial value before BSA solution addition; Δ*NTU*_2_ = difference between the NTU value registered after turbidity stabilization (after 5 min) and the initial value before BSA solution addition.

**Table 3:** Phenolic profile of the tested oenotannin solutions. Values are expressed as mg/g of oenotannin powder (mean ± standard deviation). On each column, different letters indicate significant differences among values at *p* ≤ 0.05. Mean values of condensed tannins, gallic hydrolizable tannins and ellagic hydrolizable tannins are also reported in the last three rows of the table.

Tannin Code	Gallic Acid ^1^	Castalagin ^1^	(+)-Catechin ^2^	(−)-Epicatechin ^2^	Ellagic Acid ^1^	Epicatechin Gallate ^2^	Procyanidin ^2^	Dimer Procyanidin ^2^	Trimer Procianidin ^2^	Polymers ^1^
SDS	1.77 ± 0.03 a	nd	45.68 ± 3.16 c	41.79 ± 2.43 c	nd	3.28 ± 0.14 a	27.50 ± 0.47 b	75.96 ± 0.40 b	8.12 ± 0.40 a	48.85 ± 1.06 h
SDD	13.06 ± 0.20 f	nd	175.70 ± 12.16 d	130.89 ± 7.60 e	nd	62.91 ± 2.74 c	61.35 ± 1.04 c	215.70 ± 0.65 c	13.15 ± 0.65 c	37.34 ± 0.81 g
QBS	7.98 ± 0.12 c,d	nd	10.24 ± 0.71 a,b	6.01 ± 0.35 a	nd	nd	nd	nd	nd	69.22 ± 1.50 j
QBD	13.63 ± 0.20 f	nd	3.39 ± 0.23 a	1.79 ± 0.10 a	nd	nd	nd	nd	nd	93.52 ± 2.03 k
MIM	1.41 ± 0.02 a	nd	4.30 ± 0.30 a	1.79 ± 0.10 a	nd	nd	nd	nd	11.58 ± 0.58 b	19.51 ± 0.42 d
PRU	27.39 ± 0.41 i	12.42 ± 0.65 b	15.74 ± 1.09 b	5.50 ± 0.32 a	2.02 ± 0.15 a,b	3.53 ± 0.15 a	nd	nd	nd	31.36 ± 0.68 f
CIT	29.85 ± 0.45 j	16.09 ± 0.84 d	nd	nd	1.67 ± 0.13 a	2.15 ± 0.99 a	nd	nd	nd	25.70 ± 0.56 e
THS	4.41 ± 0.07 b	nd	9.47 ± 0.66 a,b	22.19 ± 1.29 b	nd	22.37 ± 0.97 b	nd	nd	nd	4.59 ± 0.10 a
THD	9.24 ± 0.14 e	nd	13.48 ± 0.93 b	96.25 ± 5.59 d	nd	353.01 ± 15.36 d	10.49 ± 0.18 a	2.81 ± 0.16 a	nd	55.20 ± 1.20 i
GAS	34.32 ± 0.52 k	nd	nd	nd	nd	nd	nd	nd	nd	180.30 ± 3.91 n
GAD	83.45 ± 1.25 o	nd	nd	nd	15.41 ± 1.18 e	nd	nd	nd	nd	110.90 ± 2.41 m
TAR	65.75 ± 0.99 n	nd	nd	nd	nd	nd	nd	nd	nd	265.97 ± 5.77 o
MIR	14.62 ± 0.22 g	nd	nd	nd	nd	nd	nd	nd	nd	97.22 ± 2.11 l
CHS	36.36 ± 0.55 l	24.90 ± 1.30 e	nd	nd	1.97 ± 0.15 a,b	nd	nd	nd	nd	10.97 ± 0.24 b
OFN	20.11 ± 0.30 h	16.68 ± 0.87 d	nd	nd	2.35 ± 0.18 a,b	nd	nd	nd	nd	14.43 ± 0.31 c
OFT	7.36 ± 0.11 c	16.37 ± 0.85 d	nd	nd	2.49 ± 0.19 b	nd	nd	nd	nd	26.85 ± 0.58 e
OAN	7.79 ± 0.12 c,d	8.35 ± 0.44 a	nd	nd	4.68 ± 0.36 c	nd	nd	nd	nd	19.37 ± 0.42 d
OAT	8.14 ± 0.12 d	8.39 ± 0.44 a	nd	nd	6.24 ± 0.48 d	nd	nd	nd	nd	20.98 ± 0.46 d
OFE	14.80 ± 0.22 g	14.12 ± 0.74 c	nd	nd	2.17 ± 0.17 a,b	2.91 ± 0.13 a	nd	nd	nd	21.05 ± 0.46 d
OEE	37.92 ± 0.57 m	25.03 ± 1.31 e	nd	nd	2.01 ± 0.15 a,b	0.55 ± 0.02 a	nd	nd	nd	14.10 ± 0.31 c
Mean values of condensed tannins	12.08	14.25	34.75	38.27	1.84	74.54	33.11	98.15	10.95	42.81
Mean values of gallic hydrolizable tannins	61.17	nd	nd	nd	15.41	nd	nd	nd	nd	185.72
Mean values of ellagic hydrolizable tannins	18.39	16.26	nd	nd	3.13	1.73	nd	nd	nd	28.12

^1^ expressed as gallic acid equivalent; ^2^ expressed as (+)-catechin equivalent; nd: compound not detected.

**Table 4:** Asymptotic experimental *ORp* values of the oenotannin solutions at 48 h (*ORp_t48_*) versus both Ag/AgCl and SHE (+208 mV); *ORp* variations of the oenotannin solutions (Δ*ORp_t48_*) with respect to the model wine.

Tannin Code	*ORp_t48_* (mV vs. Ag/AgCl)	*ORp_t48_* (mV vs. SHE)	Δ*ORp_t48_* (mV)
SDS	108.4 ± 3.8	316.4 ± 11.2	20.7
SDD	78.8 ± 2.8	286.8 ± 10.2	−9.0
QBS	75.6 ± 2.7	283.6 ± 10.1	−12.2
QBD	72.5 ± 2.6	280.5 ± 10.0	−15.3
MIM	135.8 ± 4.8	343.8 ± 12.2	48.1
PRU	62.6 ± 2.2	270.6 ± 9.6	−25.2
CIT	61.3 ± 2.2	269.3 ± 9.6	−26.5
THS	130.5 ± 4.6	338.5 ± 12.0	42.8
THD	131.5 ± 4.7	339.5 ± 12.0	43.8
GAS	210.3 ± 7.5	418.3 ± 14.8	122.6
GAD	139.0 ± 5.1	347.0 ± 12.5	55.7
TAR	81.1 ± 3.0	289.1 ± 10.3	−4.6
MIR	86.6 ± 3.1	294.6 ± 10.4	−1.4
CHS	114.6 ± 4.1	322.6 ± 11.4	26.9
OFN	74.1 ± 2.6	282.1 ± 10.0	−13.7
OFT	70.2 ± 2.5	278.2 ± 9.9	−17.6
OAN	58.6 ± 2.1	266.6 ± 9.5	−29.2
OAT	53.7 ± 1.9	261.7 ± 9.3	−34.1
OFE	51.1 ± 1.8	259.1 ± 9.2	−36.7
OEE	210.0 ±7.5	418.0 ± 14.8	122.3
Model wine	87.8 ± 3.1	295.8 ± 10.5	0.0

**Table 5:** Characteristics (mean ± standard deviation) of the MIX 1 and MIX 2 oenotannin solutions; *AP* = antioxidant property by DPPH• assay; *A_420_* and *A_520_* = absorbance at 420 and 520 nm, respectively; L*, a*, b* and Δ*E* = CIELab coordinates; *ORp_t48_* = asymptotic experimental *ORp* values both versus Ag/AgCl and versus SHE (+208 mV); Δ*ORp_t48_* = *ORp* variations of the oenotannin solutions with respect to the model wine.

Tannin Code	Total Phenols Estimation (*w*/*w* %) ^1^	Turbidity (Δ*NTU*_1_) ^2^	Turbidity (Δ*NTU*_2_) ^2^	*AP*%	*A_420_*	*A_520_*	L*	a*	b*	Δ*E*	*ORp_t48_* (mV vs. Ag/AgCl)	*ORp_t48_* (mV vs. SHE)	Δ*ORp_t48_* (mV)
MIX 1	37.90 ± 0.02	334 ± 2	380 ± 3	89.12 ± 0.03	0.453 ± 0.001	0.119 ± 0.001	99.00 ± 0.00	−0.33 ± 0.03	3.13 ± 0.01	3.14	144.0 ± 5.1	352.0 ± 12.5	56.2
MIX 2	20.90 ± 0.01	108 ± 1	92 ± 1	90.86 ± 0.02	0.104 ± 0.000	0.049 ± 0.000	99.40 ± 0.01	−0.08 ± 0.02	1.08 ± 0.02	1.31	27.2 ± 1.0	235.2 ± 8.6	−60.6
Model wine	-	-	-	-	-	-	100.52 ± 0.05	−0.02 ± 0.03	0.40 ± 0.01	0.00	87.8 ± 3.1	295.8 ± 10.5	0.0

^1^ estimated as gallic acid equivalent/g; ^2^ Δ*NTU*_1_: difference between the NTU value at the time of BSA addition and the initial value before BSA solution addition; Δ*NTU*_2_: difference between the NTU value registered after turbidity stabilization (after 5 min) and the initial value before BSA solution addition.

**Table 6:** Polyphenol composition of the analyzed MIXs tannins in model wine solution (1 g/L). Values are expressed as mg/g of oenotannin powder (mean ± standard deviation). On each column, different letters indicate significant differences among values at *p* ≤ 0.05.

Tannin Code	Gallic Acid ^1^	Castalagin ^1^	(+)-Catechin ^2^	(−)-Epicatechin ^2^	Ellagic Acid ^1^	Epicatechin Gallate ^2^	Procyanidin ^2^	Dimer Procyanidin ^2^	Trimer Procyanidin ^2^	Polymers ^1^
MIX 1	12.94 ± 0.19 b	14.36 ± 0.75	5.28 ± 0.37 a	4.66 ± 0.27 a	3.77 ± 0.29	nd	13.19 ± 0.22 b	14.06 ± 0.81 a	0.22 ± 0.01 a	45.32 ± 0.98 b
MIX 2	5.07 ± 0.08 a	nd	35.12 ± 2.43 b	39.31 ± 2.28 b	nd	60.77 ± 2.64	12.60 ± 0.21 a	29.44 ± 1.69 b	1.38 ± 0.07 b	22.75 ± 0.49 a

^1^ expressed as gallic acid equivalent; ^2^ expressed as (+)-catechin equivalent; nd: compound not detected.

## Data Availability

The data presented in this study are available in the article.
